# Health Disparities in Czechia and Portugal at Country and Municipality Levels

**DOI:** 10.3390/ijerph16071139

**Published:** 2019-03-29

**Authors:** Michala Lustigova, Dagmar Dzurova, Claudia Costa, Paula Santana

**Affiliations:** 1 Department of Social Geography and Regional Development, Faculty of Science, Charles University, 128 43 Prague, Czech Republic; michala.lustigova@gmail.com; 2 Centre of Studies in Geography and Spatial Planning, University of Coimbra, 3004-530 Coimbra, Portugal; claudiampcosta@gmail.com (C.C.); paulasantana.coimbra@gmail.com (P.S.)

**Keywords:** population health, health determinants, health outcomes, health inequalities, Czechia, Portugal

## Abstract

This article investigates the health outcomes and determinants between two different European populations, Portuguese and Czech, on two hierarchical levels: country and metropolitan area. At first, the decomposition method of age and cause of death were compared on the country level, and then health was examined based on a factor analysis at the municipality level of Prague and Lisbon. The results clearly indicate problematic diabetes mortality among the Portuguese population, and especially in the Lisbon Metropolitan Area, and confirm the dominant role of circulatory mortality and cancer mortality among Czech, especially the Prague population. The social and economic deprivations were revealed as the major drivers for both metropolitan areas, although with differences between them, requiring interventions that go beyond the health sector.

## 1. Introduction

Life expectancy continues to rise in all European Union (EU) countries, but mortality level and cause-specific patterns of particular populations show different stages of health transition [1,2]. The female life expectancy at birth in EU countries reached 83.3 years in 2015, while that for males reached 77.9 years. However, significant differences in life span could be found on a country level. For females the lowest life expectancy at birth (LE0) was recorded in Bulgaria (78.7 years) and the highest in Spain (85.7 years), thus the difference across European populations was reaching 7 years for females. For males, the range was even higher (10.7 years), and the life expectancy at birth varied from 69.7 years in Latvia to 80.4 years in Sweden.

During the first half of the 20th century, all EU countries experienced similar patterns in mortality, characterized by a sharp decline in infectious diseases. In 1950, in general, the northern and the western parts of Europe had higher life expectancy than the Mediterranean and eastern regions [3]. Since the middle of the 1960s, cancer and cardiovascular diseases (CVD) have increased and became the prominent causes of death, and mortality started to develop differently between the western and the eastern parts of Europe. While mortality started sharply declining in Western Europe since the 1970s, especially due to new advances in the treatment of CVD, Eastern Europe, then governed by communist regimes, which had been progressing very fast in the area of infectious disease (until the mid-1960s), but were totally unable to follow the new pathway adopted by West in coping the cardiovascular diseases and some types of cancer [3]. Eastern Europe was until the end of 1980s characterized by cardiovascular crises contributing to high mortality levels and life expectancy stagnation [4]. This considerable difference in mortality trends between Western and Eastern Europe is known as the East–West mortality gap [3,5,6]. Meanwhile, the Mediterranean countries have succeeded in catching up with the northern and western countries, and nowadays belong to leaders in life expectancy in Europe.

The collapse of communist regimes at the end of the 1980s and the rapid transition from a socialist economic system to a market economic system in the Eastern European countries contributed to additional divergence in the East–West mortality gap. However, some countries, such as Czechia, experienced rapid improvement in health conditions [7,8] and a belated cardiovascular revolution (decrease in CVD mortality) took place. Others were struck by deep mortality crises and a sharp increase in mortality (e.g., Baltic states). The initial deterioration in the level of mortality was replaced in the late 1990s by the improvement; however, this turmoil brought an extra deepening of differences in mortality profiles between European populations. It will take some time before the mortality level of these countries will catch up with that of the West. However, the trends show development in the right direction.

While there is a clear East–West gap in mortality due to the circulatory system diseases, the regional distribution of cancer mortality is more mixed. Among males, the higher rates are in Central and Eastern European (CEE) countries, but the situation among females is not as uniform. Furthermore, cancer is surpassing CVD as the leading cause of death in many European populations [9]. This transition from CVD to cancer being the leading cause of death was first observed in 1998 in France and has since appeared in 11 other countries in Europe, including Portugal [9].

**Figure 1:**
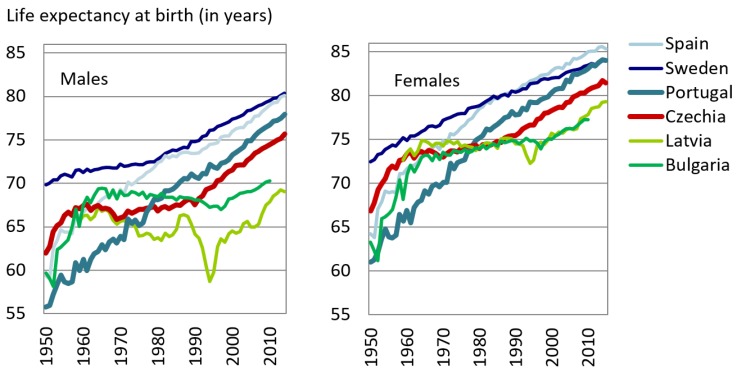
Trends in life expectancy in selected European countries, 1950–2015. Note: Sweden and Spain show the maximum male and female life expectancy, respectively, at birth within EU countries. On the other hand, Latvia and Bulgaria show the minimum male and female life expectancy, respectively, at birth within the EU countries. Data source: Human Mortality Database. University of California, Berkeley (USA), and Max Planck Institute for Demographic Research (Germany). Available at www.mortality.org or www.humanmortality.de (data downloaded on 10 March 2019).

The Portuguese population health profile was one of the worst health profiles in Europe in the 1950s. During the last six decades, Portugal has experienced a steep increase in life expectancy at birth (21 years in males and 23 years in females), and is above the European average (EU28) and approaching countries such as Sweden in female life expectancy [10]. In contrast, the health of the Czech population in the 1950s was comparable to Western European countries, but the stagnation during the communist era drastically reduced the lifespan increase. During the transformation period, the Czech population also witnessed enormous improvement in health; however, its health is still behind the European average (Figure 1).

Analyzing two different populations allows us to compare an association between health determinants and health outcomes in countries at different stages of their socioeconomic, demographic, or epidemiologic developments and compare them. Czechia is a representative of former communist countries in Eastern Europe, and since the beginning of the 1990s, has undergone a fundamental transition to a market-oriented society. Despite the improvements in living conditions, certain population groups still face material deprivation and social instability [11]. In contrast, Portugal is a former fascist country in Southern Europe, and from the middle of the 1980s, with its entry in the European Union, Portugal experienced considerable economic and social development, which was slowed down by the economic crisis in 2008. Nonetheless, Portugal has one of the most unequal income distributions in Europe [12,13]. Specifically, poverty levels are high and there are still great differences in health status between social groups and regions [12,14]. For instance, according to Santana [14], people in lower socio-economic circumstance experience higher rates of common mental health disorders; physical ill-health like coronary heart disease, chronic bronchitis, and other respiratory diseases; and musculoskeletal diseases.

Considering the population health profile of both countries, revealed by the EURO-HEALTHY Population Health Index, the health outcomes index reveals a similar pattern, especially regarding morbidity [15]. However, the health determinants index reveals two countries with different patterns, with Czechia performing better than Portugal. Socioeconomic profiles of the Portuguese and Czech population are quite different, with a low level of education and high level of unemployment being representative for Portugal, while a high level of education and low level of unemployment are more characteristic for Czechia. Meanwhile, as almost all EU capital regions, the regions where Prague and Lisbon are located present better population health profiles than the other regions in Czechia and Portugal, respectively [15]. On a metropolitan area (MA) level, Lisbon presents lower values in health determinants, but both selected metropolitan areas present lower scores in health outcomes in European comparisons [16].

Individuals and subpopulations from different backgrounds, social groups, or countries experience different levels of health. Among public health researchers, mainly unjust and preventable health inequalities are in focus. Likewise, the geographical setting plays an important role in shaping the health. Concept of space and place helps us to understand the different ways in which geography can affect health [17]. We concentrate more on place in this article, which refers to membership in an administrative unit (state, municipality district, etc.) as we want to focus on the direct studying of the area themselves as a local social and physical environment.

The United Nations recognizes the key role of reducing health-based inequalities through reducing inequality within and among countries (Goal 10) and through making cities inclusive, safe, resilient, and sustainable (Goal 11) to achieve a sustainable development [18]. This general trend to improve macroeconomic and living conditions and extend life expectancy values needs to consider details that reveal the different levels of improvement in health status between the following regions: (1) between countries [19]; (2) within countries [15]; (3) between urban and rural areas [20]; and (4) within cities [16,21]. Socioeconomic inequalities are increasingly recognized as an important public health issue, although their role in the leading causes of mortality in smaller intra-urban areas in Europe has not been fully evaluated [22]. Borrell et al. [23] showed that socioeconomic inequalities in health tend to be more pronounced in more urbanized areas (where disadvantaged and poor populations are concentrated in marginalized neighborhoods and deprived areas), and that urban areas have certain specific characteristics that can influence the populations’ health and should be a target of specific policies. For this reason, it is important to promote intersectoral work within different partners of urban governance [24]. Similarly, Marí-Dell’Olmo et al. [22] detected spatial socioeconomic inequalities for most causes of mortality studied, although these inequalities differed markedly between cities, being more pronounced in Northern and Central-Eastern Europe. Costa et al. [16] found that municipalities with worse health determinants scores tend to also perform worse on health outcomes. Samoli et al. [25] study revealed that worse air quality is typically encountered in deprived European urban areas. These authors conclude that analyzing health-related data at the level of the municipality can identify the association between health determinants and health outcomes. While those studies tried to identify general patterns and considered that processes in the cities were ongoing in a similar way, they have heterogeneous socioeconomic and epidemiologic contexts. Mitsakou et al. [26] assessed the impact of key environmental risk factors and urban environmental determinants on public health and found that they are different between European metropolitan areas. Therefore, similar mortality profiles may be due to different health determinants.

This paper identifies whether the major drivers from two different countries and metropolitan areas with similar mortality profiles are the same. A cross-national study was implemented to analyze different dimensions of population health, country-level mortality differences between the Portuguese and Czech populations by age and the main causes of death, and the health determinant in the municipality units of the metropolitan areas of Lisbon and Prague.

## 2. Data and Method

Two analyses were performed: the initial analysis—cause of death decomposition method—focused on a country level, and the other analysis—factor analysis—focused on municipality level on four causes of death and eight health determinants.

### 2.1. Areas of Interest

(A) Country Level

Data characterized trends for total mortality in Czechia and Portugal were obtained from the Human Mortality Database [27]. The data from the WHO mortality database [28] were further used for the detailed analyses of mortality structured according to age, sex, and cause of death.

Life expectancy at birth (LE0) has been widely used for analyzing the differences in populations’ health. However, LE0 does not give information about the structure or reasons causing these differences. Consequently, this work involves the decomposition of life expectancy by age-cause-specific mortality. The technique shows the differences in cause-specific mortality in each age group among two populations, i.e., the technique shows which causes of death and which age groups cause the difference and to what extent. The Pollard’s actuarial method of decomposing life expectancy was used to estimate the contributions of different age groups and disease-specific causes of death simultaneously to the changes in life expectancy [29,30]. The following five specific groups of causes of death were analyzed: circulatory system diseases, respiratory disease, cancer, diabetes, and external causes of death. All other causes of death were merged in the group called others.

**Figure 2:**
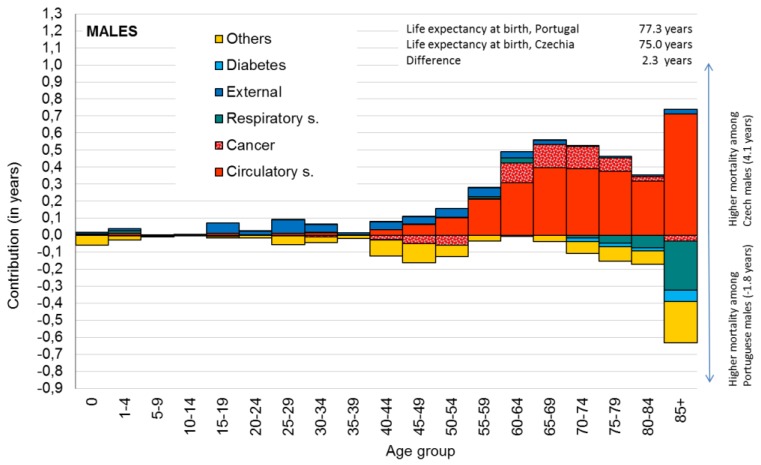
Contribution of age-specific mortality for selected groups of causes to differences in male life expectancy at birth, Portugal vs. Czechia, 2012. Data source: Human Mortality Database. University of California, Berkeley (USA), and Max Planck Institute for Demographic Research (Germany). Available at www.mortality.org or www.humanmortality.de (data downloaded on 13 March 2017). WHO mortality database. (March 2017).

The results of decomposition for the year 2012 are displayed graphically in Figure 2A,B where each column represents the contribution of each age group (x-axis), the y-axis shows the extent of this contribution, and colors are displayed to show the contributions of the selected main causes of death to LE0. Values above zero mean higher mortality among the Czech population, while values below zero mean higher mortality among the Portuguese population. 

(B) Municipality Level

The database of the EURO-HEALTHY project H2020 was used, and data on the municipality level were collected using invited focal points of each metropolitan area (Costa et al., 2019). The dataset contained aggregated data for Lisbon and Prague metropolitan areas at a municipal level (Local Administrative Unit—level 2). The Lisbon Metropolitan Area is the largest settlement in Portugal (2011: 2.82 million people) and consists of 18 areas called municipality units (administrative districts). Similarly, the metropolitan area of Prague is the largest settlement in Czechia and is divided into 57 areas. For our study, only Prague’s units with more than 5000 inhabitants were included in the analysis (N = 29 units), to reduce the bias due to small numbers in the case of mortality. Units involved in the analysis still represent 1.19 million inhabitants of Prague (in 2011). The study period was 2009–2013 for mortality outcomes for both sexes and the census year 2011 for health determinants.

### 2.2. Variables: Health Outcomes and Determinants

On the level of municipality units, two key sets of variables were used; one set described the cause-specific mortality (as health outcomes), and the second set described health determinants in multiple dimensions (as health determinants).

The first set of health outcomes was composed of four selected cause-specific mortality standardized death rates (SDRs) per 100,000 inhabitants. The four SDRs were death from diseases of the circulatory system diseases (ICD10 I00–I99), death from diseases of the respiratory system diseases (ICD10 J00–J99), death from cancer/malignant neoplasms (ICD10 C00–C97), and death from diabetes (ICD10 E10–E14). The selection of those causes of death was based on the results of the decomposition method as they caused the main differences between the lengths of life in Czechia and Portugal.

The second set of health determinants describes four selected areas of concern, which are highly associated with health inequality, as shown in *Atlas of Population Health in European Union Regions* [15]: (i) economic environment, (ii) social environment, (iii) built environment, and (iv) demographic change (Table 1).

Factor analysis was used to identify latent variables underlying health outcomes and determinants between the Lisbon and Prague units. This analysis shows correlated variables by means of a smaller number of latent variables or factors. The factor loading correlates between the factors and particular health outcomes and determinants. Each factor also produces the factor score for each municipality district, and the cut-off of 0.10 was chosen by authors in our interpretation of factors. 

The data based on municipality level contain 12 indicators of health outcomes and determinants and were analyzed using factor analysis (principal component, varimax rotation), and factors with variability over 20% separately for Lisbon and Prague. The identifiable factors using the criterion, the eigenvalue greater than one (EVG1) rule, and the factors were retained for rotation. Also, the output from the analysis was the rotated factor loadings, which are the correlations between the variable and the factor that uncovers the municipality structure of health outcomes and health determinants.

## 3. Results

### 3.1. Country Level: Epidemiological Profile of Czech and Portuguese Population in 2012

**Figure 3:**
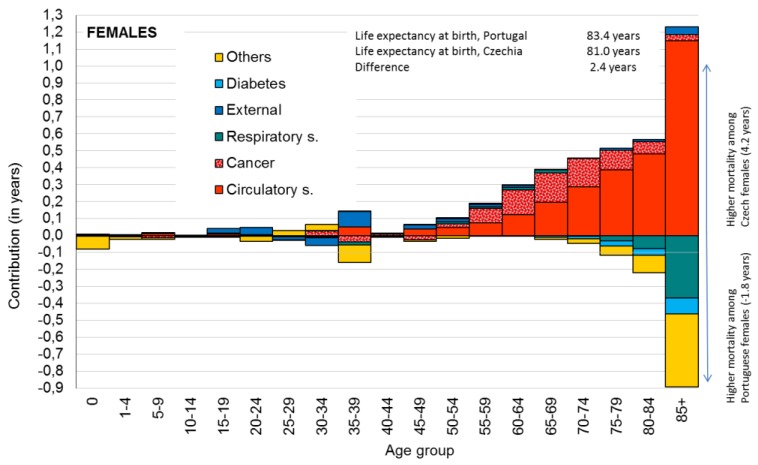
Contribution of age-specific mortality for selected groups of causes to differences in female life expectancy at birth, Portugal vs. Czechia, 2012. Data source: Human Mortality Database. University of California, Berkeley (USA), and Max Planck Institute for Demographic Research (Germany). Available at www.mortality.org or www.humanmortality.de (data downloaded on 13 March 2017). WHO mortality database. (March 2017).

Differences between the Portuguese and Czech populations in life expectancy at birth in 2012 were 2.28 years for males and 2.39 years for females. These differences in LE0 were analyzed using the decomposition method, and the contributions of age groups and selected causes of death are presented in Figure 2 and Figure 3. Positive values in the figure indicate higher mortality among the Czech population, and negative values indicate higher mortality among the Portuguese population.

Figure 2 shows visually that the main differences were in higher mortality due to circulatory system diseases (positive values) among Czech males. The sum of the positive contributions was 4.1 years and the sum of negative contributions was −1.8 years, which represents the overall difference in LE0 (2.3 years). The highest contribution according to age was found for older age groups above 55 years with the largest contribution at age 65–69 years where it was more than half a year (0.518 years). The highest contribution based on the cause of death was identified for circulatory system diseases (2.956 years). Other positive contributions (i.e., higher mortality among Czech males) were observed for external cause (0.547 years) and cancer (0.291 years). Negative contributions (i.e., higher mortality among Portuguese males) was identified for respiratory system diseases (−0.358 years) and diabetes (−0.120 years). Also, the highest contribution according to age and cause of death was observed for the oldest age group (85+) and circulatory system disease (0.712 years).

Figure 3 shows a similar structural pattern for female populations with more visible differences among the elderly. The sum of the positive contributions was 4.2 years and the sum of the negative contributions was −1.8 years, representing the overall differences in LE0 (2.4 years). The highest contribution according to the age among females was found to be similar to males for older age groups.

The contribution according to the cause of death was again positive (i.e., higher mortality among Czech females) for circulatory system diseases (2.843 years), cancers (0.745 years), and external causes (0.239 years). Also, the negative contributions (e.g., higher mortality among Portuguese males) were identified for respiratory system diseases (−0.430 years) and diabetes (−0.187 years).

### 3.2. Municipality Level: Prague and Lisbon

These four selected health outcomes present, on average, more than 900 and around 770 deaths per 100,000 inhabitants in Prague and Lisbon municipality units, respectively. Even the difference in LE0 between the countries in 2012 was more than 2.3 years, and the difference in LE0 on the metropolitan level (1.3 years) was nearly half the country LE0 difference.

Table 2 describes basic statistics of selected variables (four health outcomes, eight health determinants) for Prague and Lisbon municipality units of metropolitan areas. The differences in health between the total Czech and Portuguese population were mainly caused by higher mortality of circulatory system diseases among the Czech population (Figure 2 and Figure 3). A similar pattern was also visible on a municipality level. In Prague MA, the average SDR due to diseases of the circulatory system was 545 deaths per 100,000 inhabitants. In contrast, in Lisbon, the average SDR was 353 deaths per 100,000 inhabitants. The minimum value among Prague’s municipality units was nearly the same as the maximum among Lisbon’s municipality units (417 and 442 deaths per 100,000 inhabitants, respectively). Similarly, as the decomposition revealed higher mortality due to respiratory system diseases (RSD) and diabetes among the Portuguese population, minimal values of both causes of death are higher in Lisbon MA than the maximum values in Prague MA.

The unemployment rates (both youth and long-term) were more than two-times larger in Lisbon MA than in Prague MA (11.6% vs. 4.8% and 3.5% vs. 0.6%, respectively). Selected educational variables describe higher education in Prague MA, where 83.4% of the population attained upper secondary and tertiary education, while only 38.3% attained this level of education in Lisbon MA. Similarly, the proportion of the population with lower than secondary education was 38.9% in Lisbon MA while the proportion of the population with lower than secondary education in Prague MA was only 5.1%. Foreigners represent 15% of inhabitants in Prague and 6% in Lisbon. Both MAs were more attractive for non-EU nations; in Prague, they represented 10% of inhabitants, and in Lisbon, they represented 5%. The compared MAs differ in the number of inhabitants and in population density, where in Prague MA it was nearly two times higher than in Lisbon MA.

Two factors were included for each municipality because the variance was over 20% for each case. The first factor explained 33.4% of the total variance in the case of Lisbon and 24.2% of the total variance for the case of Prague. The cumulative variance explained by two factors was 59.6% and 46.4% for Lisbon and Prague, respectively.

**Figure 4:**
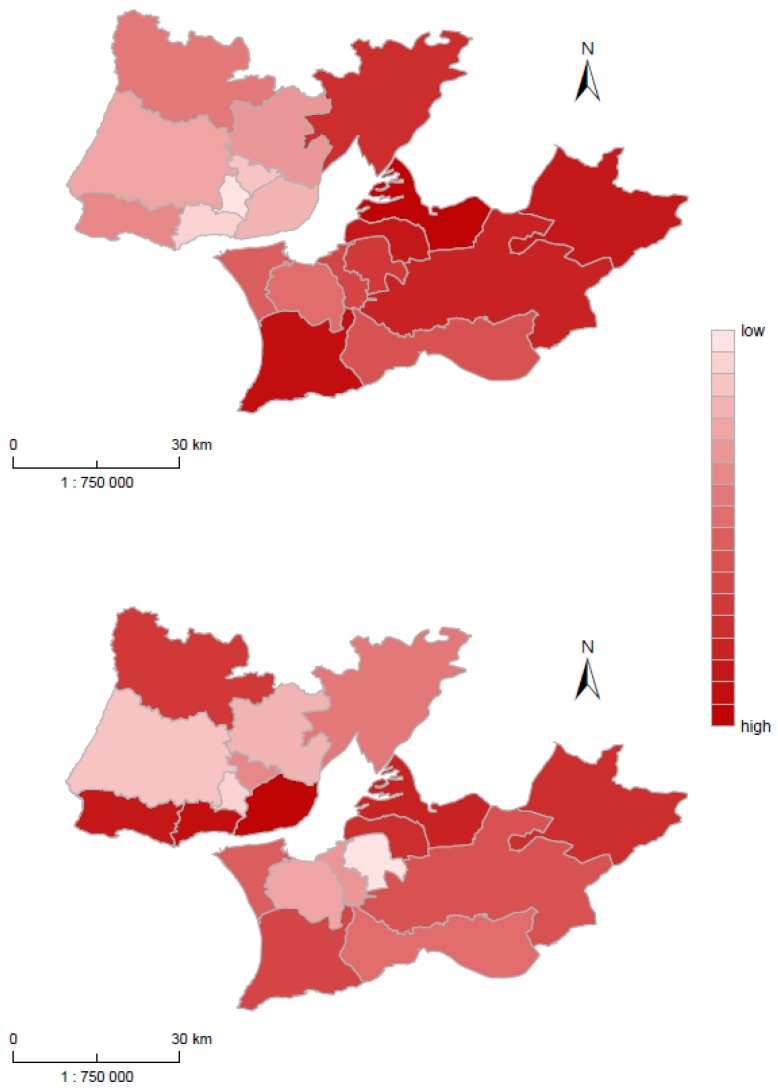
(**A**) High mortality & social and housing deprivation factor score in Lisbon MA. (**B**) Low mortality & economic, social, and housing prosperity factor score in Lisbon MA.

Table 3 presents the factor loadings for the first two factors for Lisbon municipalities, and Figure 4A,B show the maps of factor scores for both factors. For Lisbon MA, the Factor 1 is called “High mortality & social and housing deprivation“. This factor aggregates a high indicator for all health outcomes in a positive direction, especially diabetes mortality (*r* = 0.833) and circulatory mortality (*r* = 0.794), and population density in a negative direction (*r* = −0.842). In fact, it was the highest correlation of any health outcome with any factor for both cases. This result clearly indicates problematic diabetes mortality among the Portuguese population, and especially in Lisbon. Also, from the set of health determinants, low population density and low proportion of immigrants from non-EU countries were heavily dependent on Factor 1. This result confirms the association between the diabetes mortality and geographic dimension with municipalities out of the central part with high diabetes mortality. Figure 4A reveals two areas within Lisbon MA, North and South, which predominantly corresponded to two former NUTSIII regions: Grande Lisboa in the North and Peninsula de Setúbal in the South. This factor was heavily concentrated in Peninsula de Setúbal, where rural municipalities with lower population density were present. In contrast, the lowest factor scores were concentrated in the Grande Lisboa, which was characterized by the low level of mortality. This low mortality was associated with high population density.

Factor 2 for Lisbon MA is labelled “Low mortality & economic, social, and household prosperity”. It was associated with positive health determinants (highest values of factor loadings) such as high educational level, high level of housing condition, and a high proportion of the population born in EU28 countries. Cancer mortality was highly correlated with this factor (*r* = 0.389). Compared to Factor 1, Factor 2 did not show a clear geographical pattern (Figure 4B). The highest scores occurred on the south of Grande Lisboa subregion (Lisbon, Cascais, Oeiras) in municipalities with the lowest mortality level associated with a higher proportion of tertiary-educated population. In contrast, the lowest value of factor loading occurred in Moita in the North municipality of Peninsula de Setúbal with the lowest proportion of the tertiary-educated population.

**Figure 5:**
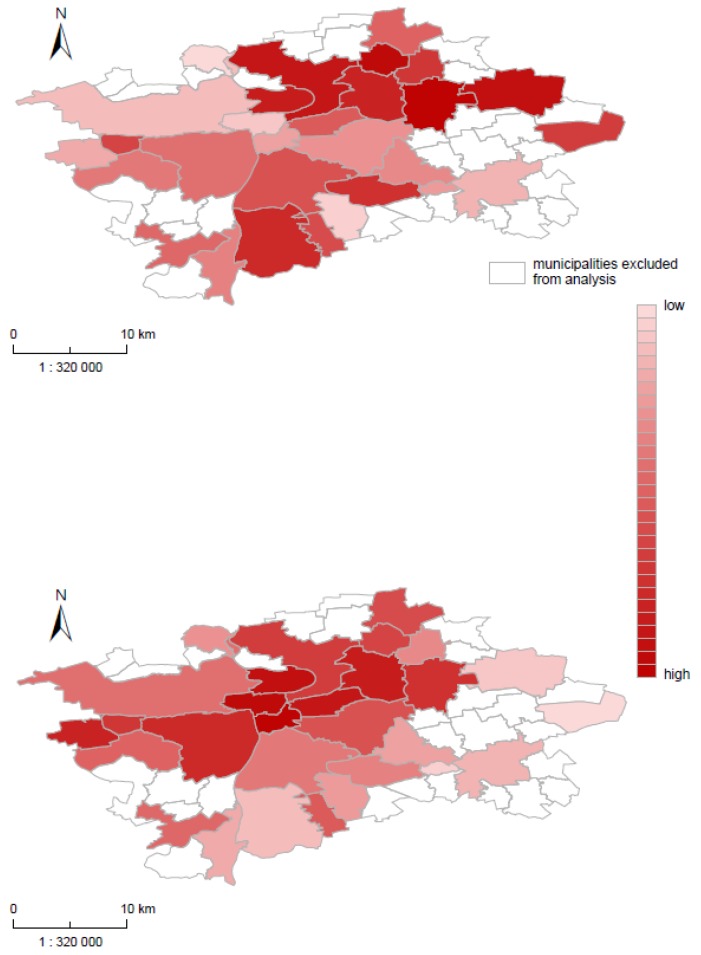
(**A**) High circulatory mortality & economic and social deprivation factor score in Prague MA. (**B**) High mortality & social and housing deprivation factor score in Prague MA.

Table 4 represents factor loadings for Prague MA, and Figure 5A,B shows the map of factor scores for Factors 1 and 2. Factor 1 is labelled “High circulatory mortality & economic and social deprivation,” and the highest health outcome loading is circulatory mortality (*r* = 0.632). In addition, the highest health determinants were both unemployment indicators and low education. This factor confirmed the dominant role of circulatory mortality among Czech, especially the Prague population. All other health outcomes are very weakly correlated with Factor 1, and this factor was heavily concentrated in the north-east part of Prague, especially in municipalities Prague 14, Prague 18, and Prague 20 (high positive values of factor score) (Figure 5A). In these municipalities, the highest circulatory mortality was combined with the highest unemployment. These regions were thus not attractive for EU28 citizens. In contrast, the lowest factors scores were concentrated in the region from the center to the north-west of Prague, namely Prague 1 (center), Prague 6 and Prague-Suchdol. In these regions, a low level of circulatory mortality was associated with low unemployment and a high proportion of EU28 citizens. 

Factor 2 is labelled “High mortality & social and housing deprivation” and was correlated with all health outcomes, especially with cancer (*r* = 0.474) and circulatory mortality (*r* = 0.430). A high mortality was associated with a low education level and low housing conditions, and with a higher proportion of immigrants from EU and non-EU countries. Compared to Factor 1, the high values of factor scores were placed in the central part of Prague with the highest population density (characterized by a higher level of mortality due to cancer and circulatory system diseases) (Figure 5B). The population structure of this area was also characterized by the lower educational level.

## 4. Discussion

### 4.1. Principal Findings and Strengths of the Study

This article investigates the health outcomes and determinants between two different European populations, Portuguese and Czech, on two hierarchical levels. First, the decomposition method of age and cause of death were compared on the country level, and then health was examined based on factor analysis at the municipality level of Prague and Lisbon.

On the country level, the differences between the Portuguese and Czech populations in life expectancy at birth in 2012 were 2.28 years for males and 2.39 years for females. The differences in life expectancy between these two populations were mainly caused by higher mortality of circulatory system diseases among the Czech population and higher mortality due to respiratory system diseases and diabetes among the Portuguese population. This mortality profile from both countries was also identified in previous studies regarding the country [10,31,32,33] and metropolitan areas [22,34]. According to our results, the mortality of circulatory system diseases was two times higher in Prague’s population than in Lisbon. In contrast, a higher level of mortality was found for respiratory system diseases and diabetes among the Lisbon population (two times and three times, respectively), but the level of mortality of these causes were significantly lower.

Considering the metropolitan areas study, the results identified that in each metropolitan area, different dimensions (health determinants) play a role. For Factor 1 (Lisbon: Low mortality & economic, social and household prosperity; Prague: High circulatory mortality & economic and social deprivation), the different level of population health was only associated with the mortality of diseases of the circulatory system in Prague, but with all the selected causes of deaths in Lisbon, with the highest impact on diabetes mortality. The high circulatory mortality in Prague’s units was associated with the economic and social environment dimensions, i.e., with economic and social deprivation. Such a finding confirmed the results from Lustigova et al. [33], which identified education as the strongest determinant of cardiovascular mortality among the Czech population. In addition, results revealed that these deprived areas in Prague were not attractive for immigrants, who were mainly from EU28 countries. In Lisbon’s units, a high level of diabetes mortality was found in municipalities with the lowest population density and low proportion of immigrants from non-EU28 countries. Problematic diabetes mortality among Portuguese and Lisbon populations was analyzed using two ecological studies at country and metropolitan area levels [31,34]. In Portugal, the highest mortality due to diabetes was found in more rural/less urbanized areas with the highest index of sociomaterial deprivation [31,34]. Then, respiratory mortality was related to selected health determinants only in Lisbon’s units. A recent study on Lisbon MA found a positive correlation between NO_2_ exposure and population density, population ageing, and population born in non-EU28 countries [25]. Respiratory health is also affected by climate changes [35], especially heat waves, drought conditions, and wildfires (smoke emissions). The Portuguese population was more affected by these events than the Czech population, where wildfires are very rare. Thermal comfort (cold in winter) is another key indoor factor that might affect health, especially for the elderly in Portugal [36,37,38].

Factor 2 from both cities (Lisbon: Low mortality & economic, social and household prosperity; Prague: High mortality & social and housing deprivation) reveals an opposite dependence regarding cancer mortality. While in Prague’s units, the higher mortality was associated with a lower level of education and a lower level of housing condition; in contrast, in Lisbon, the association was the opposite. Cancer mortality was associated with a higher level of education, housing conditions, and a high proportion of immigrants from EU28 countries (but a low proportion of immigrants from non EU28 countries). These findings were already confirmed in Marí-Dell’Olmo et al. [22], where lung cancer mortality was positively associated with deprivation in Northern European cities, but this association was negative in Southern European cities, including Lisbon. This opposite pattern in this study [22] is explained by different stages of the behavioral epidemic, mainly a smoking epidemic. In the final stage of a smoking epidemic, the prevalence of smoking is higher among the population with lower socioeconomic status, while in the initial phases of a smoking epidemic, smoking was a privilege of persons with higher socioeconomic position [39]. Incidence of lung cancer reflects the smoking habits and smoking prevalence 30 years ago. We assume that at that time, the smoking was more frequent among higher socioeconomic groups, especially among Portuguese females. According the data from European Health Interview Survey—EHIS 2014 [40], even nowadays the lowest smoking prevalence is observed among females with primary education, and among Portuguese males, the prevalence of smoking is at the same level for all educational groups.

These results call for intervention at a local level. For Lisbon, policies must be addressed in order to reduce social inequities within the metropolitan area by investing in interventions that are able to help finding a job; to promote lifelong learning; to support the requalification of housing (e.g., improvement of energy efficiency); and to boost health literacy activities. Similarly, for Prague, policies are needed to reduce social inequalities and improve access to sustainable employment opportunities. Additionally, transforming the built environment and promoting the development of green spaces are needed to improve overall health and well-being.

### 4.2. Strengths and Limitations

This study has several notable strengths. First, it includes an analysis of two hierarchical levels. Second, for the first time, the combination of decomposition methods on country level and ecological study approach on a municipality level was used. Third, the results of decomposition were applied as background for further ecological study analysis. In contrast, the limitation of this study represents the comparability of areas of interest (size) of municipality units. As metropolitan areas and municipality units differ significantly in population size and density; thus, the analysis was done separately for Lisbon and Prague.

We are aware that the health outcomes are strongly correlated by individual-level socioeconomic status, but several studies have shown an increased risk of mortality for people living in more deprived neighborhoods, regardless of individual-level education, occupation, or income [41,42]. Even a neighborhood effect is not controlled for due to a data limitation for individual-level socioeconomic status in this study; nonetheless, in general, the effect persists even after adjusting for personal social characteristics [43].

## 5. Conclusions

European countries and cities face different demographic, social, and economic challenges. The results of this study support the conclusion that health inequities varied between groups of people within cities and between cities. The key health determinants of health inequalities were not similar and depend on the historical development of the city and the current setting of the social support system. Furthermore, the study revealed different sources of urban health inequality between Lisbon and Prague.

Health inequalities can be, at municipality units level, reduced by public policies such as e.g., improvements in housing and building standards (e.g., affordable heating, ventilation, rehousing and renovation reducing the risk of falls and injuries), ensuring of local service availability (e.g., high-quality green and open spaces, and subsidised childcare), or lowering of speed limits and separation of pedestrians, cyclists and vehicles. Improvements in the daily living conditions in the administrative unit can promote the population health. Our findings provide evidence for policy makers at lower administrative unit and may therefore contribute to the implementation of policy programs reducing health inequalities.

## Figures and Tables

**Table 1:** Health Determinants indicators.

Area of Concern	Dimension	Indicator *
Economic environment	Employment	Youth unemployment rate (%)
		Long-term unemployment rate: 12 months or more (%)
Social environment	Education	Population aged 25–64 with lower secondary education attainment (%)
		Population aged 25–64 with upper secondary or tertiary education attainment (%)
Built environment	Housing conditions	Number of rooms per person
	Population density	Population density (inhabitants/km^2^)
Demographic change	Migration	Population born in EU28 countries, excluding the reporting country (%)
		Population born in non-EU28 countries (%)

Note: * For detailed information on indicators see Santana et al. [15], pp. 243–249.

**Table 2:** Descriptive statistics of health outcomes and health determinants for Prague and Lisbon metropolitan areas (2009–2013).

Indicator/Variable	Prague (N = 29 Municipality Units)				Lisbon (N = 18 Municipality Units)			
	Minimum	Maximum	Mean	Std. Deviation	Minimum	Maximum	Mean	Std. Deviation
**Health Outcomes**								
Deaths from diseases of the circulatory system *	417.3	675.5	544.7	63.3	276.3	442.1	353.2	38.5
Death from cancer *	245.2	339.2	287.5	25.2	236.6	280.7	257.5	14.6
Death from diseases of respiratory system *	17.3	77.0	60.0	14.5	85.48	157.7	113.3	20.5
Death from diabetes *	4.1	27.4	13.5	5.1	29.57	78.14	47.9	12.9
**Health Determinants**								
Youth unemployment rate (%)	2.9	6.1	4.8	0.81	7.7	16.3	11.6	2.47
Long term unemployment rate (%)	0.2	1.1	0.6	0.22	2.3	5.0	3.5	0.76
Population with lower secondary education (%)	3.1	7.1	5.1	0.92	25.7	46.7	38.9	5.54
Population with upper secondary or tertiary education (%)	67.1	88.5	83.4	4.72	29.2	52.5	38.3	5.98
Housing condition (Average number of rooms per person)	1.1	1.5	1.3	0.08	1.7	2.0	1.8	0.08
Population density (inhabitants/km^2^)	564.7	11,747	3781.4	3078.49	135.5	7370.6	2038.8	2235.60
Population born in EU28 countries (%)	3.0	9.9	5.0	1.38	0.2	2.4	0.9	0.60
Population born in non-EU28 countries (%)	4.5	15.8	10.1	2.86	2.0	9.5	5.1	2.00

Note: * standardized death rate per 100,000 inhabitants. Data source: All variables are taken from EURO-HEALTHY project.

**Table 3:** Lisbon municipalities factor loadings.

**Component**	**Indicators**	**Factor 1** **High Mortality & Social and Housing Deprivation**	**Factor 2** **Low Mortality & Economic, Social, and Housing Prosperity**
		**Factor Loading**	**Factor Loading**
**Health Outcomes**	Deaths from diseases of the circulatory system	**0.794**	−0.148
	Death from diseases of respiratory system	**0.760**	−0.162
	Death from diabetes	**0.833**	
	Death from cancer	**0.428**	**0.389**
**Health Determinants**	Youth unemployment rate	0.239	−0.320
	Long term unemployment rate	−0.151	**−0.447**
	Population with lower secondary education	**0.394**	**−0.863**
	Population with upper secondary or tertiary education	**−0.397**	**0.878**
	Population density	**−0.842**	
	Housing condition		**0.851**
	Population born in EU28 countries	0.252	**0.628**
	Population born in non-EU28 countries	**−0.753**	**−0.436**

Notes: Bold > ±0.350; Factor loadings represent the correlations between health outcomes and health determinants and two factors. All factor loadings over 0.10 are reported in the table.

**Table 4:** Prague municipalities factor loadings.

**Component**	**Indicators**	**Factor 1** **High Circulatory Mortality & Economic and Social Deprivation**	**Factor 2** **High Mortality & Social and Housing Deprivation**
		**Factor Loading**	**Factor Loading**
**Health Outcomes**	Deaths from diseases of the circulatory system	**0.632**	**0.430**
	Death from diseases of respiratory system		0.259
	Death from diabetes	−0.149	0.284
	Death from cancer		**0.474**
**Health Determinants**	Youth unemployment rate	**0.861**	
	Long term unemployment rate	**0.784**	
	Population with lower secondary education	**0.845**	−0.146
	Population with upper secondary or tertiary education		**−0.759**
	Population density	0.112	**0.612**
	Housing condition	−0.238	**−0.795**
	Population born in EU28 countries	**−0.484**	**0.505**
	Population born in non EU28 countries	−0.146	**0.549**

Notes: Bold > ±0.350; Factor loadings represent the correlations between health outcomes and health determinants and two factors. All factor loadings over 0.10 are reported in the table.
